# New Medical School

**Published:** 1851-09

**Authors:** 


*New Medical School.*—The N. Y. Med. Gazette states that measures are on foot to establish a new Med. School in the city of New York, in connection with the New York Hospital.

